# Elite Swimmers’ Training Patterns in the 25 Weeks Prior to Their Season’s Best Performances: Insights Into Periodization From a 20-Years Cohort

**DOI:** 10.3389/fphys.2019.00363

**Published:** 2019-04-10

**Authors:** Philippe Hellard, Marta Avalos-Fernandes, Gaelle Lefort, Robin Pla, Inigo Mujika, Jean-François Toussaint, David B. Pyne

**Affiliations:** ^1^Research Department, French Swimming Federation, Pantin, France; ^2^CREPS Bordeaux-Aquitaine, Bordeaux, France; ^3^Centre d’Etudes des Transformations des Activités Physiques et Sportives, EA-3832, Faculté des Sciences du Sport, Université de Rouen, Mont-Saint-Aignan, France; ^4^Institut National de Recherche en Informatique et en Automatique SISTM, Bordeaux, France; ^5^INSERM, UMR 1219, University of Bordeaux, Bordeaux, France; ^6^École Nationale de la Statistique et de l’Analyse de l’Information (ENSAI), Bruz, France; ^7^Department of Physiology, Faculty of Medicine and Odontology, University of the Basque Country, Leioa, Spain; ^8^Exercise Science Laboratory, School of Kinesiology, Faculty of Medicine, Universidad Finis Terrae, Santiago, Chile; ^9^EA 7329, Paris Descartes University, Sorbonne Paris Cité University, Paris, France; ^10^Centre d’Investigation en Médecine du Sport, Hôpital Hôtel-Dieu, AP-HP, Paris, France; ^11^Research Institute for Sport and Exercise, University of Canberra, Canberra, ACT, Australia

**Keywords:** training distribution, progressivity, competitive performance, swimming, latent class mixed models

## Abstract

**Background:**

This study investigated the periodization of elite swimmers’ training over the 25 weeks preceding the major competition of the season.

**Methods:**

We conducted a retrospective observational study of elite male (*n* = 60) and female (*n* = 67) swimmers (46 sprint, 81 middle-distance) over 20 competitive seasons (1992–2012). The following variables were monitored: training corresponding to blood lactate <2 mmol⋅L^-1^, 2 to ≤4 mmol⋅L^-1^, >4–6 mmol⋅L^-1^, >6 mmol⋅L^-1^, and maximal swimming speed; general conditioning and maximal strength training hours; total training load (TTL); and the mean normalized volumes for both in-water and dryland workouts. Latent class mixed modeling was used to identify various TTL pattern groups. The associations between pattern groups and sex, age, competition event, Olympic quadrennial year, training contents, and relative performance were quantified.

**Results:**

For the entire cohort, ∼86–90% of the training was swum at an intensity of [La]_b_ ≤ 4 mmol⋅L^-1^. This training volume was divided into 40–44% at <2 mmol⋅L^-1^ and 44–46% at 2 to ≤4 mmol⋅L^-1^, leaving 6–9.5% at >4–6 mmol⋅L^-1^, and 3.5–4.5% at >6 mmol⋅L^-1^. Three sprint TTL patterns were identified: a pattern with two long ∼14–15-week macrocycles, one with two ∼12–13 week macrocycles each composed of a balanced training load, and one with a single stable flat macrocycle. The long pattern elicited the fastest performances and was most prevalent in Olympic quadrennials (i.e., 4 seasons preceding the 2004, 2008, and 2012 Olympic Games). This pattern exhibited moderate week-to-week TTL variability (6 ± 3%), progressive training load increases between macrocycles, and more training at ≤4 mmol⋅L^-1^ and >6 mmol⋅L^-1^. This fastest sprint pattern showed a waveform in the second macrocycle consisting of two progressive load peaks 10–11 and 4–6 weeks before competition. The stable flat pattern was the slowest and showed low TTL variability (4 ± 3%), training load decreases between macrocycles (*P* < 0.01), and more training at 4–6 mmol⋅L^-1^ (*P* < 0.01).

**Conclusion:**

Progressive increases in training load, macrocycles lasting about 14–15 weeks, and substantial volume of training at intensities ≤4 mmol⋅L^-1^ and >6 mmol⋅L^-1^, were associated with peak performance in elite swimmers.

## Introduction

To achieve the fastest competition performances, elite coaches periodise athletes’ training loads over multi-year and annual training programs (Turner, 2011; Mujika et al., 2018). Periodization is the purposeful sequencing of training units (long-, medium-, and short-term training cycles and sessions) designed to produce cumulated adaptations that peak during major competitions (Mujika et al., 2018). Current theoretical models of annual periodization (Issurin, 2016; Mujika et al., 2018) argue for cyclical or wave variation of the training load, evolving from the beginning of each general training mesocycle toward increasingly specific and intensive overload periods before the taper. During the taper phase, reduced training volume while maintaining intensity helps potentiate the adaptations while enabling athletes to recover from the detrimental effects of physiological stress (Mujika et al., 1996, 2018). The social environment, training regimes, and competition all place heavy demands on elite athletes, pushing them to the limits of adaptation (Sandbakk and Holmberg, 2017; Mujika et al., 2018). Strategically alternating phases of overload and recovery can limit performance decrements through injury, overtraining, and detraining (Turner, 2011; Issurin, 2016; Mujika et al., 2018). The progressive increase in training loads (swimming volume and/or intensity, strength training and minor competitions) from one macrocycle to another over an Olympic cycle, and throughout an athlete’s career, should ensure that the training stimulus persists to yield new adaptations and progress (Kraemer and Ratamess, 2004; Platonov, 2006; Lyakh et al., 2014; Issurin, 2016).

Observational studies of small cohorts of high-level athletes are the main source of data for periodization models for World-class and Olympic athletes in cross-country skiing (Tønnessen et al., 2014; Sandbakk and Holmberg, 2017; Solli et al., 2017), cycling (Schumacher and Mueller, 2002), rowing (Fiskerstrand and Seiler, 2004), and running (Esteve-Lanao et al., 2005). These models typically divide the annual cycle into two to four periods (macrocycles): general preparation periods (high training volume at intensities corresponding to powers or velocities associated with blood lactate concentration ([La]_b_) ≤ 4 mmol⋅L^-1^ and strength training) alternating with more specific periods at higher intensity, ending with the intense competitive phases (Schumacher and Mueller, 2002; García-Pallarés et al., 2010). In several sports like cross-country skiing (Fiskerstrand and Seiler, 2004; Sandbakk and Holmberg, 2017; Solli et al., 2017), orienteering (Tønnessen et al., 2015), and rowing (Fiskerstrand and Seiler, 2004), the general preparation periods are long (18–24 weeks) and the training load increases progressively until stabilization. Specific training periods may be shorter (8–12 weeks) and reach a peak load followed by a decrease just before the competitive phase. In other sports like cycling (Schumacher and Mueller, 2002), triathlon (Mujika, 2014), and kayaking (Issurin, 2016), the annual periodization is characterized by shorter macrocycles (12–16 weeks) made up of general, specific and competitive mesocycles lasting 2–6 weeks. The periodization methods of elite endurance athletes who have progressed throughout their careers display increases in total training load, volume, training intensity and number of competitions (Esteve-Lanao et al., 2005; Tønnessen et al., 2014; Solli et al., 2017). Reports on annual periodization for rowing (Fiskerstrand and Seiler, 2004; Guellich et al., 2009), triathlon (Mujika, 2014) and cycling (Schumacher and Mueller, 2002) generally show an increase in the total load in the summer as opposed to winter, whereas for cross-country skiing, orienteering and athletics (Tønnessen et al., 2014, 2015), only the intensity increases in the second part of the season.

Regarding the training intensity distribution, the pyramidal model (high proportion of training at [La]_b_ ≤ 2 mmol⋅L^-1^ and a progressive decrease in the proportions at intensities between 2 and 4 mmol⋅L^-1^, and >4 mmol⋅L^-1^) appears to apply to most elite endurance athletes (Stöggl and Sperlich, 2015). However, differences in the proportions of training intensities are evident among sports, indicating that intensity distribution depends as much on sport-specific techniques as the duration and energetic profile of the competitive event (Stöggl and Sperlich, 2015; Mujika et al., 2018). For example, although rowing competitions are among the shortest in terms of duration (7–8 min), international rowers (Guellich et al., 2009) perform 85–90% of their training at moderate intensity ([La]_b_ < 2.5 mmol⋅L^-1^) and only 3% at severe and extreme intensity (>4 mmol⋅L^-1^). For elite cross-country skiers, the training contents (80–90% at <2.5 mmol⋅L^-1^, 3–5% at 2–4 mmol⋅L^-1^, 5–8% at >4 mmol⋅L^-1^, 10% training for strength and speed) are quite similar irrespective of the competition distance (i.e., 1.3–1.8 km for sprint events and 30 or 50 km for mid-distance events) (Sandbakk and Holmberg, 2017; Solli et al., 2017). Lastly, in weight-bearing sports (triathlon, cross-country and marathon running), the intensity zone distributions are similar (about 70–80% at <2.5 mmol⋅L^-1^, 20–30% at 2–4 mmol⋅L^-1^, and 5–10% at >4 mmol⋅L^-1^), even though the competition events are typically longer (Billat et al., 2001; Esteve-Lanao et al., 2005). Studies comparing the training characteristics of world-class athletes versus lower-level athletes (Guellich et al., 2009; Sandbakk and Holmberg, 2017) have reported ∼15–30%, higher training volumes especially in low intensity zones at [La]_b_ ≤ 2 mmol⋅L^-1^, and higher amounts of speed training and strength training. Elite training has changed over the past 40 years, with most studies reporting changes in total training volume coupled with a higher proportion of endurance training at low intensity (Stöggl and Sperlich, 2015). We sought to quantify the training of elite swimmers over a 20 years period encompassing multiple Olympic Games and World Championships.

In swimming, observational studies (research not interfering with training scheduling or regimens) have shown that the intensity distribution has shifted from a pyramidal model with a high proportion of aerobic training in the 1990s (over 70% of training at [La]_b_ < 2 mmol⋅L^-1^) (Mujika et al., 1996) toward models with a high proportion of training between 2 and 4 mmol⋅L^-1^ in the 2000s (35–50%) (Avalos et al., 2003; Hellard et al., 2006). These studies, however, report a relatively small proportion of training above 4 mmol⋅L^-1^ (9–12%). Several elite coaches have shared their annual periodization programs for selected World and Olympic champions (Pyne and Touretski, 1993; Maglischo, 2003; Barnier, 2012; Vergnoux, 2014), mainly in case study format. Most programs follow periodization models close to those in other endurance sports: organization of the season into two to four macrocycles of 8–15 weeks, division of each macrocycle into mesocycles (or blocks) of 2–5 weeks, and progression from general to specific training. These reports from coaches at international technical symposia (Maglischo, 2003) indicate that Olympic and World champion middle-distance swimmers (200–400 m) follow a predominantly pyramidal model, with 55–70% of training at [La]_b_ < 2 mmol⋅L^-1^ and 30–40% between 2 and <4 mmol⋅L^-1^ (Maglischo, 2003). In sprint swimming (50–100 m), the literature (Maglischo, 2003) reveals two types of distribution in champion sprinters and Olympic medallists, with the first showing an annual volume of ∼2000–2500 km with a consistent proportion (∼90%) of training at ≤4 mmol⋅L^-1^ (Pyne and Touretski, 1993), and the second a smaller annual volume of ≤1500 km of training at [La]_b_ ≤ 2 mmol⋅L^-1^ accounting for more than 70% of the volume and training ≥4 mmol⋅L^-1^, tending toward 15% (Barnier, 2012). However, no study has yet detailed the evolution of periodization patterns and training characteristics (volume and intensity distributions) over multiple Olympic cycles, and their relation to competition performance, in a large cohort of elite swimmers.

The aim of this study was to investigate the patterns and characteristics of training profiles in elite swimming to gain insight into long-term periodization. This work is based on a large cohort of elite French swimmers followed for a median of 3 years. We analyzed the training loads quantified over the 25 weeks preceding the best annual performance of 127 national and international swimmers. The profiles were related to factors such as the year in the Olympic cycle, event specificity (technique, distance, and sex), age, performance level, and training contents. This information will inform the planning and evaluation of elite-level swimming training.

## Materials and Methods

### Study Sample

The detailed training programs of 127 nationally- and internationally ranked male and female swimmers and their competition performance times were recorded over 20 competitive seasons. All swimmers trained in one of two national training centers. Swimmers were excluded if they had a chronic pathology (illness and/or injury) requiring medical treatment, had missed training for 4 weeks or more, or were taking medication known to affect immune function or inflammation. Only swimmers who met the inclusion criteria were selected. The experimental study was conducted in accordance with the Declaration of Helsinki. The study was approved by the institutional review board of the host site (Institut National du Sport, de l’Expertise et de la Performance), and all participants gave written informed consent. Written informed consent was obtained from the parents for all participants under the age of 16 years old. In addition, individual swimmer-seasons with very slow performances or noticeably irregular training programs were excluded. The study sample therefore comprised 289 individual swimmer-seasons corresponding to 127 swimmers followed for a median of 3 years (range 1–11).

Sixty swimmers were male and 67 were female, aged between 15 and 30 years. Four swimmers specialized in 50 m and 42 in 100-m events (sprinters); 52 swimmers specialized in 200 m and 29 in 400 m and longer distances (middle-distance). Forty-six sprint swimmers specialized (although not exclusively) in front crawl, 27 in breaststroke, 18 in butterfly and 14 in backstroke. Five middle-distance swimmers specialized in breaststroke, 37 in front crawl, 11 in butterfly, 10 in backstroke and 18 in individual medley. The swimmers competed in 7 ± 4 (mean ± SD) competitions per season.

### Training and Performance Measures

All performance (P) times in seconds at official competitions in Olympic size 50 m pool were recorded. To account for year-to-year changes in competition conditions – for example, period of full-body polyurethane-based swimsuits in 2007–2009 – the performance times are expressed relative to the mean of the 10 best world performance times (M_10_WP) in a given year for a given sex, stroke, and distance: Pr $(\%)=\frac{100(\text{P}-{\text{MML:M}}_{\text{10}}\text{WP})}{{\text{MML:M}}_{10}\text{WP}}$, where Pr indicates a swimmer’s relative performance (the lower the Pr value, the closer a swimmer’s performance to the current M_10_WP). Only each swimmer’s best performance of the season was taken into account.

The intensities for quantifying the swim workouts at the two national centers were determined using the method of Mujika et al. (1996) under the supervision of the French Swimming Federation. An incremental test to exhaustion was performed at the beginning of each season (repeated and adjusted 4 times per season) to determine the relationship between blood lactate concentration and swimming speed. Each subject swam 6 m × 200 m at progressively higher percentages of their personal best competition time, culminating in a maximal effort on the sixth and final swim. Lactate concentration was measured in capillary blood collected from the fingertip during the 1-min recovery period separating the 200 m swims (Mujika et al., 1996). All swimming sessions were categorized into five intensity levels: I1: below 2 mmol⋅L^-1^, I2: from 2 to 4 mmol⋅L^-1^, I3: above 4–6 mmol⋅L^-1^, I4: above 6 mmol⋅L^-1^, and I5: maximal swimming speed. The speeds corresponding to each intensity level were then corrected to account for the swimming distance and rest intervals using Olbrecht et al. (1985). For a female World champion and Olympic medalist, typical I1 and I2 training sets were, respectively, 30 m × 100 m with 15 s rest swum in 1:10 min:s with [La]_b_= 1.5 mmol⋅L^-1^, and 20 m × 100 m with 40-s rest swum in 1:06 min:s with [La]_b_= 3.8 mmol⋅L^-1^. A typical I3 training set for the same swimmer was 12 m × 100 m with 25-s rest swum in 1:03 min:s with [La]_b_= 5.8 mmol⋅L^-1^. A typical I4 training set was 8 m × 50 m with 30-s rest swum in a mean 28.6 s with [La]_b_ = 7.6 mmol⋅L^-1^. In-water workouts were quantified in meters per week at each intensity level.

Strength training included dryland workouts at maximal strength training (ST) (1–6 repetitions, 80–100% of 1 repetition maximum: 1RM) and general conditioning (GC). Strength training was quantified in minutes of active exercise per week (Avalos et al., 2003). To simplify and reduce the number of independent variables, we used the following practical notations: ∼moderate-to-heavy intensity (MHI; < the sum of I1 and I2, [La]_b_ ≤ 4 mmol⋅L^-1^), ∼severe intensity (SI; I3, [La]_b_ > 4–6 mmol⋅L^-1^), ∼extreme-intensity (EI; the sum of I4 and I5, [La]_b_ > 6 mmol⋅L^-1^), GC), and ST (Hellard et al., 2017). The categories MHI, SI, and EI were based on swimming speeds and blood lactate measurements following the literature classifications (Dekerle et al., 2005; Fernandes and Vilas-Boas, 2012). The upper limit for heavy intensities was defined as the speed at the maximum lactate steady state, which has been shown to correspond to [La]_b_ = 3.3 ± 2.5 mmol⋅L^-1^ (Dekerle et al., 2005). The upper limit for severe intensities was defined as the lowest swimming speed at which peak oxygen uptake is reached, with the lowest [La]_b_ reported in the literature of ∼6.5–7.0 mmol⋅L^-1^ (Fernandes and Vilas-Boas, 2012). For each swimmer and each season, training for the 25 weeks preceding the best performance of the season was quantified.

In this study, we analyzed training intensities and workouts with four approaches:

(1) Absolute training loads. Each individual swimmer’s mean of absolute training volumes were calculated over the 25 weeks preceding the season’s best performance. In-water workouts were quantified weekly in meters at each intensity level and summarized through the individual mean over the 25 weeks. Dryland workouts were quantified weekly in minutes of active exercise and averaged over the 25 weeks.
(2) Progressivity. To investigate progressivity in the training profiles, we compared the differences in each individual’s mean over the first and second halves of the 25 weeks training period.
(3) Distribution. Each individual swimmer’s mean intensity distribution was determined. The individual’s proportion of MHI, SI and EI were calculated as the mean MHI, SI, and EI over the 25 weeks relative to the mean total in-water workout over the 25 weeks, respectively. The proportion of general conditioning was calculated as the mean of the total dryland training. The proportions of intensity distributions varied between 0 and 1 and their sum was 1, with the proportions of general conditioning and strength training in the form of compositional data (Bacon-Shone, 2011).
(4) Total training load (TTL) and variation in TTL. For each swimmer and each season, the weekly proportion of pool and dryland training were scaled as a percentage of the maximal volume measured at the same intensity level. Thus, for each swimmer, season and intensity, the values were rescaled between 0 and 100%, and the maximum 100% was achieved in at least 1 week, which facilitated comparisons across time and between swimmers. The weekly TTL calculated as the mean normalized volumes for both in-water and dryland workouts (all 5 in %) summarized the weekly training volume and intensity relative to the swimmer’s capacities (Avalos et al., 2003; Hellard et al., 2017). The training profile analyses were based on TTL. The variation in training was measured as the mean difference in TTL between two consecutive weeks (in %). This measure was used to summarize differences in variability between training profiles.

### Covariates

Several factors may affect adaptations to a training program. We considered the following covariates: age at the time of competition, sex, stroke, the quarter (phase of the season) accounting for the relative importance of the competitions (i.e., national competitions typically in the first and second quarters, and international competitions in the third), season number since entry into the study (as a marker of a swimmer’s experience), and the season of the quadrennial (post-Olympic, World Championships, pre-Olympic and Olympic seasons) accounting for the absolute importance of the competitions. We also considered two distance classes: sprint swimmers specialized in 50-m or 100-m events and middle-distance swimmers specialized in the 200-m and/or 400-m.

### Statistical Analysis

Training for the 25 weeks preceding each swimmer’s best performance of the season was used in the analyses since this was the longest period common to all subjects between the beginning and the best performance of the season. The analyses were stratified by two distance classes: sprint swimmers and middle-distance swimmers. However, there was substantial heterogeneity in the analysis of the middle-distance swimmers’ training trajectories.

First, we described the sample in terms of covariates. All analyses were stratified by the two distance classes.

Secondly, we used latent class mixed models to identify the TTL pattern groups over time. This model combines a latent class model to identify homogeneous latent groups of subjects, and a mixed model to describe the mean trajectory over time in each latent group, while taking into account the individual correlation between repeated measures. The hlme function of the lcmm R-package was used to estimate the model parameters (Proust-Lima et al., 2018). The training trajectories were described according to the number of weeks preceding the best performance of the season using spline functions without adjustment for baseline covariates. The shapes of the group-specific and subject-specific trajectories were determined by comparing models with an increasing number of parameters (1–15 knots in the spline functions of time, a diagonal or unstructured random-effect covariance matrix, and a class-specific or proportional random-effect covariance matrix). The best model among those with the same number of groups was selected using the Akaike Information Criterion. Individual class membership was not fixed, and estimated on the highest probability of belonging to a given class from a multinomial logistic model. In each run, the number of distinct classes had to be specified. The decision about the number of latent classes (1–5) was based on both the Bayesian Information Criterion and interpretation of the distinct groups.

Thirdly, we characterized the highlighted profiles in terms of demographic characteristics, swim specialty, time-variant covariates, distribution of training intensities, and relative performance. ANOVA or Kruskal–Wallis rank sum tests (if ANOVA conditions were not fulfilled) were used to determine differences in training volumes among groups. Paired *T* or Wilcoxon signed-rank tests (if *T*-test conditions were not fulfilled) were used to determine differences within each first-half and second-half pair of training volumes. MANOVAs were used to determine differences in in-water training proportions and dryland training proportions (after applying the isometric logratio transformation for compositional data) among groups (Bacon-Shone, 2011). Given the exploratory nature of this observational study, association tests between profiles and covariates were performed on the assumption that the number of profiles was correct and subjects were classified correctly, with a significance level of 0.05, and without applying multiplicity corrections.

## Results

### Training Profiles in Sprinters: Identification

**FIGURE 1:**
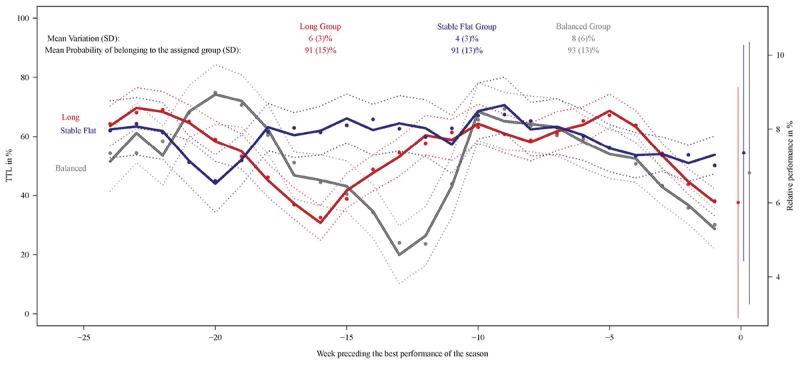
**(Left axis)**: weighted mean subject-specific predictions of TTL in % (solid circles), the observed class-specific mean evolutions weighted by the class-membership probabilities (solid lines) and their 95% confidence limits (dashed lines) by week preceding performance. Mean (SD) difference in TTL between two consecutive weeks in % per group and mean (SD) probability of belonging to the assigned group (top legend). **(Right axis)**: observed mean relative performances per group in % and standard deviations. Sprint swimmers (46 swimmers, 105 swimmer-seasons).

The total number of swimmer-seasons analyzed was 105. Three groups, each with a distinct training profile, were identified over the 25 weeks preceding the best performance of the season (Figure 1, right axis). The mean probability of belonging to the assigned group was high (≥91%), indicating very reliable classification. The Long group and the Balanced group showed two-macrocycle profiles with marked fluctuations in each macrocycle consisting of a progressive load increase in the beginning of the macrocycle, one or two load peaks lasting 1–3 weeks, and a progressive load decrease lasting 2–4 weeks corresponding to the taper period. The Stable Flat group showed a more regular pattern and a longer second cycle (approximately 16 vs. 13 weeks) with a TTL of about 60% with minor deviations during taper. Respectively, 53, 24, and 23% of the swimmer-seasons were classified into the Long, Balanced and Stable Flat groups. Among swimmers followed for more than one season, 80% did not train with the same profile.

### Training Profiles in Sprinters: Characterization

In terms of performance, training contents and distributions, sprint trajectory groups could be characterized as follows. The group corresponding to a pattern of Long macrocycles was the fastest characterized by a medium training load, medium variability, training load progressivity between the first and second macrocycles, two load peaks (highest load in each macrocycle) 21 and 5 weeks before their best performance, and an intensity distribution with a greater amount of training at [La]_b_ ≤ 4 mmol⋅L^-1^ and >6 mmol⋅L^-1^. The group corresponding to a pattern of Balanced macrocycles was characterized by a low training load, high variability, degressivity between the first and second macrocycles, two load peaks 19 and 10 weeks before the best performance, and a moderate quantity of training at >4–6 mmol⋅L^-1^. The group corresponding to a pattern of Stable Flat macrocycles was the slowest characterized by a high training load, low variability, degressivity between the first and second macrocycles, a single load peak 8 weeks before BP, and the highest quantity of training at [La]_b_ > 4–6 mmol⋅L^-1^.

The group corresponding to a pattern of Stable Flat macrocycles presented the lowest variation (Figure 1), measured as the mean difference in TTL between 2 consecutive weeks (mean variation ± SD: 4 ± 3% compared with 6 ± 3% for the Long group and 8 ± 6% for the Balanced group). The Long group showed the best performance (6.0 ± 3.1%) compared to the Balanced (6.8 ± 3.5%, *P* = 0.31) and Stable Flat (7.4 ± 2.9%, *P* < 0.05) groups, although standard deviations were high.

Tables 1, 2 presents the predominant demographic and swimming characteristics of each of the three outcome groups. The Stable Flat macrocycle pattern group was the youngest and the Balanced group the oldest (*P* < 0.01). The Balanced group was mostly composed of swimmers in the early seasons of the study period (mostly the September 1992–September 1996 Olympic cycle) and, similarly, most of the Stable Flat group was from the September 1996–September 2000 Olympic cycle. In contrast, the Long group consisted mainly of recent swimmer-seasons (mostly September 2000–September 2012) (*P* < 0.001). Post-Olympic and World Championships seasons predominated in the Long group, pre-Olympic seasons were most frequent in the Stable Flat group, and Olympic seasons were frequent in the Balanced group (*P* < 0.0001).

**Table 1:** Summary of predominant characteristics of sprint swimmers by periodization profile of training.

	Long	Stable flat	Balanced
Pattern	2 well-defined cycles, regular pattern, longer 2nd cycle.	TTL of about 60%, minor deviations during taper	2 well-defined balanced cycles
Variability	Moderate 6 (3)%	Low 4 (3)%	High 8 (6)%
Performance	Fastest	Slowest	Medium
Training volume	Low–medium	Large	Low
Progressivity	Progressivity	Intermediate degressivity	Large degressivity
Distribution	Large volume [La]_b_ ≤ 4 and >6 mmol/L.	Large volume [La]_b_ between 4 and 6 mmol/L.	Medium volume [La]_b_ ≤ 4 and > 6 mmol/L.
Peak in the second macrocycle.	5 weeks before the best performance.	8 weeks before the best performance.	10 weeks before the best performance.
Age	Intermediate	Youngest	Oldest
Quadrennials	Recent	Early	Mostly 1st quadrennial
Season	Post-Olympic and World Championship	Pre-Olympic	Olympic

**Table 2:** Trajectory groups for sprint swimmers by demographic characteristics, swim specialty and time-variant covariates.

	Trajectory groups for sprint swimmers			
	Long group	Stable group	Balanced group	*P*
Qualitative covariates: sample size (%)				
Total	56 (100)	24 (100)	25 (100)	
Gender Female	32 (57)	14 (58)	13 (52)	0.90
Male	24 (43)	10 (42)	12 (48)	
Specialty freestyle	24 (43)	11 (46)	11 (44)	0.90
Breaststroke	15 (27)	6 (25)	6 (24)	
Butterfly	9 (16)	5 (21)	4 (16)	
Backstroke	8 (14)	2 (8)	4 (16)	
Distance 50 m	5 (9)	1 (4)	1 (4)	0.70
100 m	51 (91)	23 (96)	24 (96)	
Quarter 2nd	31 (55)	11 (46)	16 (64)	0.40
3rd	25 (45)	13 (54)	9 (36)	
Season from entry in the study 1	18 (32)	7 (29)	8 (32)	0.50
2	16 (29)	10 (42)	4 (16)	
3	8 (14)	3 (12)	7 (28)	
≥4	14 (25)	4 (16)	6 (24)	
Season in quadrennial Post-Olympic	18 (32)	6 (25)	1 (4)	<0.0001
World Championship	21 (38)	5 (21)	4 (16)	
Pre-Olympic	9 (16)	10 (42)	6 (24)	
Olympic	8 (14)	3 (12)	14 (56)	
Quadrennial September 1992–September 1996	9 (16)	1 (4)	13 (52)	<0.001
September 1996–September 2000	3 (6)	15 (62)	6 (24)	
September 2000–September 2004	18 (32)	2 (8)	3 (12)	
September 2004–September 2008	17 (31)	3 (12)	3 (12)	
September 2008–September 2012	9 (16)	3 (12)	0 (0)	
Quantitative covariate: Mean ± SD				
Age at the performance date in *y*	20.6 ± 3.2	18.8 ± 2.6	21.5 ± 3.4	<0.01

*Values are sample sizes (row percentages) for categorical covariates and mean ± SD for quantitative covariates. P-values correspond to chi-squared or Fisher exact test (if chi-squared conditions not fulfilled) for categorical covariates, ANOVA or Kruskal–Wallis rank sum test (if ANOVA conditions not fulfilled) for quantitative covariates.*

**FIGURE 2:**
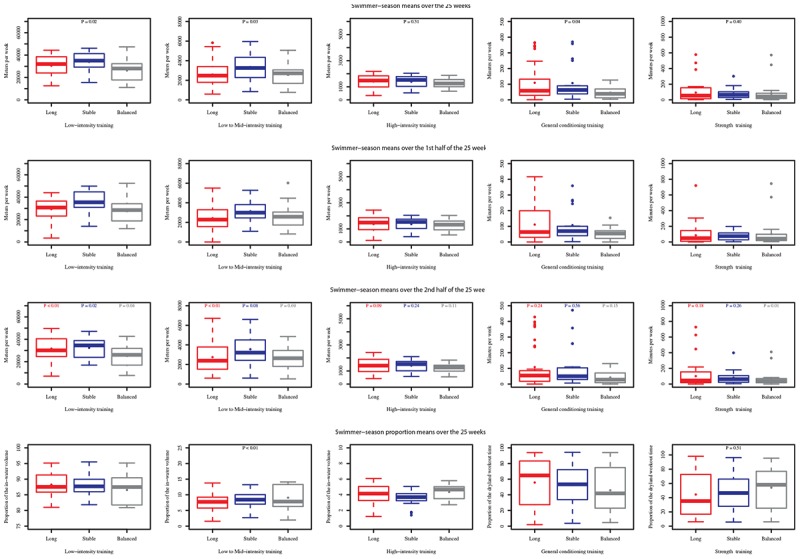
Moderate-to-heavy (1st column), Severe- (2nd column), Extreme- (3rd column) intensity training, General conditioning (4th column), and Strength training (5th column) means per swimmer-season distributions by group in sprint swimmers using box plots (solid circles indicate the mean values). Mean per swimmer-season values were calculated over the 25-week period (1st row), the 1st half of this period (2nd row) or the 2nd half of this period (3rd row). Proportion mean per swimmer-season values were calculated over the 25-week period (4th row). *P*-values determine differences in training intensities among groups (1st row), within each 1st-half and 2nd-half pair of mean measurements (3rd row), in in-water and dryland variables among groups (4th row).

Figure 2, 1st row, shows the differences between the training profile groups with respect to the volume in absolute values. With moderate-heavy and severe intensity training, the Stable Flat group had substantially greater volume than the Balanced group (moderate-heavy intensity: Balanced: 26,300 ± 9,600 m.wk^-1^ vs. Long: 30,600 ± 91 m.wk^-1^ vs. Stable Flat: 34,010 ± 9,600 m.wk^-1^, *P* < 0.05; severe intensity: Balanced: 2,550 ± 1,130 m.wk^-1^ vs. Long: 2,590 ± 240 m.wk^-1^ vs. Stable Flat: 3,350 ± 1,240 m.wk^-1^, *P* < 0.05) (both *P* < 0.05). In contrast, the Long group had the highest volume of general conditioning training (46 ± 36 vs. 109 ± 119 vs. 107 ± 116 m.wk^-1^, *P* < 0.05).

Between the first half of the studied period (the first 13 weeks, Figure 2, 2nd row) and the second half (the last 12 weeks, Figure 2, 3rd row), moderate-high intensity, severe intensity and extreme intensity volumes increased in the Long macrocycle pattern group (*P* < 0.01, *P* < 0.01, and *P* < 0.10, respectively). Conversely, moderate-heavy intensity decreased in the Stable Flat and Balanced groups (both *P* < 0.05). In the latter, strength training also decreased (*P* < 0.05).

**FIGURE 3:**
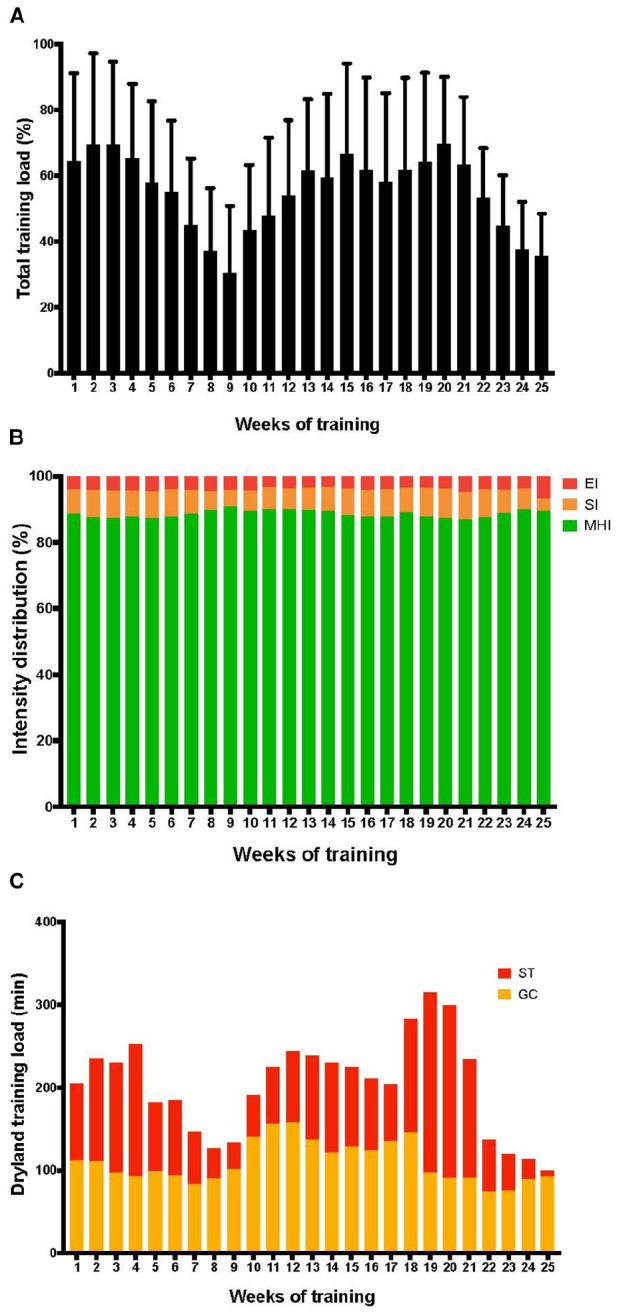
In-water and dryland intensity distribution for the fastest group. **(A)** Shows the total training load with the peak load in week 22 in the first macrocycle and in week 6 in the second macrocycle before the best performance of the season. **(B)** Shows the training intensity distribution, with moderate-to-heavy intensity (MHI, ≤4 mmol⋅L^-1^) in green, severe intensity (SI, above 4 up to 6 mmol⋅L^-1^) in orange, and extreme intensity (EI, >6 mmol⋅L^-1^) in red. Note the intensification of training in weeks 3, 4, and 5 before the best performance of the season. **(C)** Shows the distribution of dryland training, general conditioning in orange, and total strength training in red. Note the largest proportion of dryland training in weeks 5, 6, and 7 before the best performance of the season.

Figure 2, 4th row, shows the proportion of each training intensity for in-water and dryland training by group. Globally, the distribution of in-water training intensities differed among groups (*P* < 0.01). The proportion of moderate-heavy intensity training was lower in the Balanced group, the proportion of severe intensity was lower in the Long group, and the proportion of EI was lower in the Stable Flat group. Figure 3 highlights swimming and dryland intensity distribution for the fastest group.

### Training Profiles in Middle-Distance Swimmers: Identification

**FIGURE 4:**
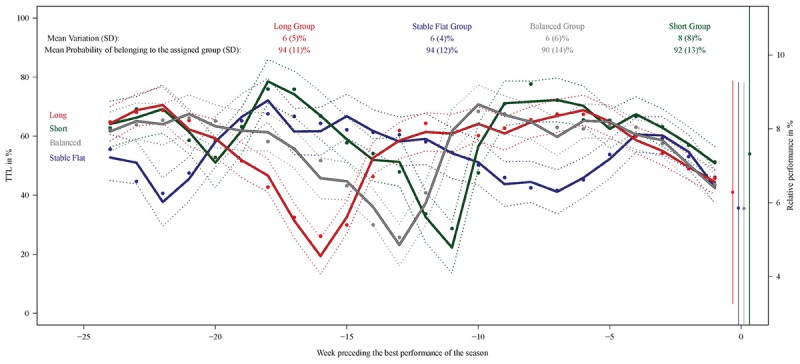
**(Left axes)**: weighted mean subject-specific predictions of TTL in % (solid circles), the observed class-specific mean evolutions weighted by the class-membership probabilities (solid lines) and their 95% confidence limits (dashed lines) by week preceding performance. Mean (SD) difference in TTL between 2 consecutive weeks in % per group and mean (SD) probability of belonging to the assigned group (top legend). **(Right axes)**: observed mean relative performances per group in % and standard deviations. Mid-distance swimmers (81 swimmers, 184 swimmer-seasons).

The total number of individual swimmer-seasons was 184. As shown in Figure 4, the mean probability of belonging to the one of the four assigned groups was high (≥90%). The Long and Balanced groups exhibited two-macrocycle profiles with well-marked intensive and tapering periods. The second macrocycle lasted about 11 weeks before the best performance for the Balanced group vs. 16 weeks for the Long group. The Short macrocycle pattern group showed a more irregular pattern than the other two with three macrocycles, with the last two lasting about 10 weeks. The fourth group, the Stable Flat macrocycle pattern group, exhibited a profile with the lowest peaks around 40% of TTL and a short taper period (progressive decrease in TTL starting 3 weeks before the best performance). Respectively, 36, 20, 25, and 19% of the swimmer-seasons were classified into the Long, Stable Flat, Balanced, and Short groups. Among swimmers followed for more than one season, 87% did not train with the same profile.

### Training Profiles in Middle-Distance Swimmers: Characterization

Table 3 presents the predominant demographic and swimming characteristics of each of the four outcome groups. The Long macrocycle pattern group presented a high swimming training volume, low dryland volume, medium variability, progressivity between the two macrocycles, two load peaks 20 and 6 weeks before the best performance, and a moderate quantity of training at [La]_b_ > 4–6 mmol⋅L^-1^. The Stable Flat macrocycle pattern group was characterized by a low volume of swimming training, a high volume of dryland training, medium variability, degressivity between the two macrocycles, two load peaks 17 and 3 weeks before the best performance, and a high amount of training [La]_b_ ≤ 4 mmol⋅L^-1^ and >6 mmol⋅L^-1^. The Balanced macrocycle pattern group showed low swimming and dryland training loads, medium variability, progressivity in TTL between the first and second macrocycles, two load peaks 20 and 9 weeks before the best performance, and a high amount of training [La]_b_ ≤ 4 mmol⋅L^-1^ and >6 mmol⋅L^-1^. Lastly, the Short macrocycle pattern group was the slowest characterized by a high training load, high variability, degressivity in TTL from the first to the second macrocycle, two load peaks 18 and 6 weeks before BP, and a greater amount of training [La]_b_ ≤ 4 mmol⋅L^-1^ and >6 mmol⋅L^-1^.

**Table 3:** Summary of predominant characteristics of middle-distance and distance swimmers by periodization profile of training.

	Long	Stable flat	Balanced	Short
Pattern	2 well-defined cycles, longer 2nd cycle	Lowest peaks ∼40% of TTL, a short taper period	2 well-defined balanced cycles	2-cycles, shorter 2nd cycle
Variability	Moderate 6 (5%)	Moderate 6 (5%)	Moderate 6 (6%)	High 8 (8%)
Performance	Medium	Second fastest	Fastest	Slowest
Training volume	High in-water, low dryland training	Low in-water, high strength training	Low in-water and dryland training	High in-water and dryland
Progressivity	Progressivity	Degressivity	Progressivity	Degressivity
Distribution	Intermediate volume [La]_b_ ≤ 4 and >6 mmol/L.	Large volume [La]_b_ ≤ 4 and >6 mmol/L.	Large volume [La]_b_ ≤ 4 and >6 mmol/L.	Large volume [La]_b_ between 4 and 6 mmol/L.
Peak in the second macrocycle.	6 weeks before the best performance.	3 weeks before the best performance.	9 weeks before the best performance.	6 weeks before the best performance.
Swimmer’s experience	Experienced	Experienced	Experienced	Inexperienced
Season	Pre- and post-Olympic	Pre- and post-Olympic	Olympic and World Championship	Pre-Olympic

**Table 4:** Trajectory groups for middle distance swimmers by demographic characteristics, swim specialty and time-variant covariates.

	Trajectory groups for Middle Distance swimmers				
	Long group	Stable group	Balanced group	Unstable group	*P*
Qualitative covariates: sample size (%)					
Total	66 (100)	36 (100)	47 (100)	35 (100)	
Gender female	25 (38)	18 (50)	20 (43)	18 (51)	0.50
Male	41 (62)	18 (50)	27 (57)	17 (49)	
Specialty freestyle	11 (46)	16 (44)	24 (51)	15 (43)	0.10
Breaststroke	2 (3)	4 (11)	2 (4)	1 (3)	
Butterfly	3 (5)	6 (17)	5 (11)	8 (23)	
Backstroke	3 (5)	6 (17)	7 (15)	8 (23)	
4 strokes	16 (24)	6 (17)	9 (19)	6 (17)	
Quarter 2nd	35 (53)	10 (28)	23 (49)	18 (51)	0.08
3rd	31 (47)	26 (72)	24 (51)	17 (49)	
Season from entry in the study 1	0 (0)	5 (14)	5 (11)	2 (6)	0.02
2	8 (12)	6 (17)	8 (17)	12 (34)	
3	10 (15)	3 (8)	5 (11)	5 (14)	
≥4	48 (73)	22 (61)	29 (62)	16 (46)	
Season in quadrennial Post-Olympic	20 (30)	8 (22)	10 (21)	8 (23)	0.01
World Championship	16 (24)	7 (19)	13 (28)	12 (34)	
Pre-Olympic	19 (29)	14 (39)	6 (13)	13 (37)	
Olympic	11 (17)	7 (19)	18 (38)	2 (6)	
Quadrennial September 1992–September 1996	5 (8)	5 (14)	7 (15)	7 (20)	0.20
September 1996–September 2000	7 (11)	6 (17)	7 (15)	11 (31)	
September 2000–September 2004	27 (41)	11 (31)	12 (26)	9 (26)	
September 2004–September 2008	18 (27)	11 (31)	12 (26)	5 (14)	
September 2008–September 2012	9 (14)	3 (8)	9 (19)	3 (9)	
Quantitative covariate: Mean ± SD					
Age at the performance date in *y*	20.8 ± 3.1	19.9 ± 3.0	21.0 ± 3.4	19.8 ± 3.1	0.20

*Values are sample sizes (row percentages) for categorical covariates and mean ± SD for quantitative covariates. P-values correspond to chi-squared or Fisher exact test (if chi-squared conditions not fulfilled) for categorical covariates, ANOVA or Kruskal–Wallis rank sum test (if ANOVA conditions not fulfilled) for quantitative covariates.*

The Short group showed the highest variation (mean variation ± SD: 8 ± 8% compared with 6% for the others, with SD 6% or smaller) (Figure 4). The relative performances in each group are also presented in Figure 4 (right axis). The Short group showed slower relative performance (mean relative performance ± SD: 7.3 ± 4.7%) than the Balanced (5.8 ± 3.4%, *P* < 0.10), Stable Flat (5.9 ± 3.4%, *P* = 0.13) and Long (6.3 ± 3.0%, *P* = 0.18) periodization groups, although standard deviations were high.

Table 4 presents the distribution of covariates by group. Pre- and post-Olympic seasons were the most frequent in the Stable Flat and Long groups. Conversely, Olympic Games and World Championships were the most represented seasons in the Balanced group. Last, pre-Olympic seasons were the most frequent in the balanced group, and this group also presented the lowest number of Olympic seasons (*P* = 0.01). In each group, more than half the observations were made in experienced swimmers, except for the balanced group, which presented the highest percentage of new entries (1 and 2 years) into the study (*P* = 0.02).

**FIGURE 5:**
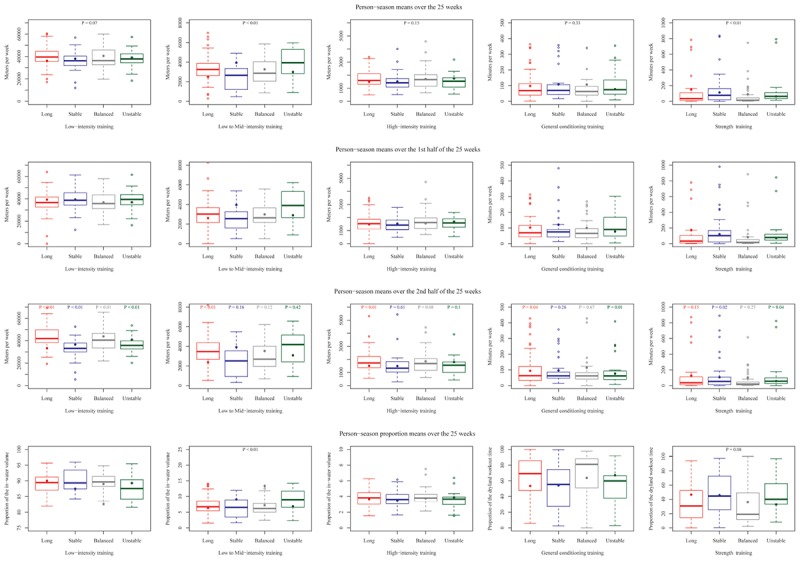
Moderate-to-heavy (1st column), Severe- (2nd column), Extreme- (3rd column) intensity training, General conditioning (4th column) and Strength training (5th column) means per swimmer-season distributions by group in mid-distance swimmers using box plots (solid circles indicate the mean values). Mean per swimmer-season values were calculated over the 25-week period (1st row), the 1st half of this period (2nd row) or the 2nd half of this period (3rd row). Proportion mean per swimmer-season values were calculated over the 25-week period (4th row). *P*-values determine differences in training intensities among groups (1st row), within each 1st-half and 2nd-half pair of mean measurements (3rd row), in in-water and dryland variables among groups (4th row).

Figure 5, 1st row, shows that for MHI the Stable Flat group had a substantially lower absolute volume than the group corresponding to a Long pattern (*P* = 0.07) and the group corresponding to the Short pattern (*P* < 0.01) (36160 ± 8420 vs. 37950 ± 6870 vs. 40480 ± 8800 vs. 39050 ± 9560 m.wk^-1^, in Stable Flat, Short, Long, and Balanced groups, respectively). SI was higher for the Short group and lower for the Stable Flat group (2490 ± 1250 vs. 3940 ± 1580 vs. 3270 ± 1310 vs. 2990 ± 1320 m.wk^-1^, in Stable Flat, Short, Long, and Balanced, respectively, *P* < 0.01). In contrast, the group corresponding to the Stable Flat pattern had the highest volume of ST training.

Between the first half (Figure 5, 2nd row) and the second half (Figure 5, 3rd row) of the studied period, MHI volumes decreased in the Short and Stable Flat groups, whereas they increased in the Balanced and Long groups (all *P* < 0.01). In the Long group, SI and EI also increased (both *P* < 0.01). EI also increased in the Balanced group (*P* = 0.08), whereas it decreased in the Short group (*P* = 0.10). GC decreased in the Short group and increased in the Long group (*P* = 0.01, *P* = 0.04), and ST decreased in the groups corresponding to the Short and Stable Flat patterns (*P* = 0.04, *P* = 0.02).

Figure 5, 4th row, shows the proportion at each training intensity by group. Globally, ∼86–90% of the training was swum at MHI and training volume was divided as follows: 42–44% at [La]_b_ < 2 mmol⋅L^-1^ and 44–46% between 2 and ≤4 mmol⋅L^-1^. The distribution of in-water and dryland training intensities differed by group (*P* < 0.01 and *P* = 0.08, respectively). The proportions of MHI and EI were lower in the Short group and higher in the Stable Flat group. The ST proportion was lower in both the Balanced and Long groups.

## Discussion

This research is the first to analyze different periodization methods and their contents over several Olympic cycles in a large cohort of elite male and female swimmers, including nine Olympic or World Championships medalists. The study period covered 20 competitive seasons, resulting in 105 swimmer-seasons for sprinters and 184 swimmer-seasons for middle-distance swimmers, with individual follow-up ranging from 1 to 11 seasons. The extent of the modeling is also indicated by the detailed analysis of the 25-week seasons, which captured the entire preparation for national competitions and the major international meet of each season.

The use of latent class mixed models provided empirical identification of three distinct training profiles in sprint swimmers and four profiles in middle-distance swimmers. We characterized these groups in terms of performance, training load contents, variability of training, progressivity and training distribution. For the 289 swimmer-seasons, ∼86–90% of the training was swum at an intensity of [La]_b_ ≤ 4 mmol⋅L^-1^. This training volume was divided into 40–44% at <2 mmol⋅L^-1^ and 44–46% at 2 to ≤4 mmol⋅L^-1^, leaving 6–9.5% at >4–6 mmol⋅L^-1^ and 3.5–4.5% at >6 mmol⋅L^-1^. Although each training profile showed a large dispersion in the performances, the group of sprint swimmers corresponding to the pattern of Long macrocycles (characterized by macrocycles lasting 15–16 weeks, medium training load, medium variability, progressivity between the two macrocycles, a load peak 4 weeks before BP, and a distribution characterized by a greater amount of training ≤4 and >6 mmol⋅L^-1^) had the fastest performances. Conversely, the sprint swimmers flowing the Stable Flat pattern and the middle-distance swimmers who followed a Short pattern (both characterized by the highest training load, degressivity between the two macrocycles, a load peak 8 and 6 weeks before BP, and a greater amount of training at >4–6 mmol⋅L^-1^) were the slowest. These outcomes support the assertion that contemporary swimming periodization should give priority to macrocycles that last 13–16 weeks, with sufficient training load variability, progressivity between the two macrocycles, a load peak ∼3–5 weeks before major competition, and the greatest portions of training at ≤4 mmol⋅L^-1^ (∼85–90%) and >6 mmol⋅L^-1^ (∼4–5%).

### Training Profiles – Distribution of Training Intensities

The distribution of training intensities in our study cohort follows the threshold model (Solli et al., 2017) with ∼86.5–89% below [La]_b_ 4 mmol⋅L^-1^ (43% below 2 mmol⋅L^-1^), 6–9.5% between 4 and 6 mmol⋅L^-1^, and 3.5–4.5% above 6 mmol⋅L^-1^. These proportions differ from those reported for other endurance sports (running, cycling, rowing, and cross-country skiing), which showed the high prevalence of a pyramidal distribution with a smaller proportion of training at intensities between 2 and 4 mmol⋅L^-1^. These observational studies of exercise-intensity distribution in endurance sports (Billat et al., 2001; Esteve-Lanao et al., 2005; Guellich et al., 2009; Sandbakk and Holmberg, 2017) reported that high-level athletes typically perform 75–80% of their training below the aerobic or ventilatory threshold (about 2 mmol⋅L^-1^) and 5–15% above the lactate threshold (about 4 mmol⋅L^-1^) (Stöggl and Sperlich, 2015). Our analysis of the swimming data highlighted a higher percentage of training in the 2–4 mmol⋅L^-1^ zone (44–46%), in contrast to the training contents for other endurance sports, which show a proportion of approximately 2–16% of training at intensities in this intensity zone. In swimming, the speeds and stroke rates corresponding to intensities <2 mmol⋅L^-1^ are much slower than those of actual competition (e.g., a female World champion performed a set of 10 m^∗^100 m corresponding to a [La]_b_ of 1.5 mmol⋅L^-1^ at a swim speed of 1.2 m.s^-1^ and a stroke rate of 25.5 s.min^-1^, whereas her competition speed was 1.82 m.s^-1^ with a stroke rate of 49 s.min^-1^). At these slower speeds corresponding to [La]_b_ ≤ 2 mmol⋅L^-1^, catch-up coordination induces intra-cyclic velocity variations and an increase in energy cost (Vilas-Boas et al., 2011). In contrast, distance per stroke and swimming efficiency have been shown as the highest around the maximal lactate steady state speed corresponding to [La]_b_ 3–5 mmol⋅L^-1^ (Dekerle et al., 2005). In summary, better technical efficiency in the 2–4 mmol⋅L^-1^ zone may be one of the reasons for the greater proportion of swimming training in these zones compared with other cyclical endurance sports (e.g., cross-country skiing, etc.).

The fastest profiles for both sprinters and middle-distance swimmers were characterized by a greater amount of training up to [La]_b_ 4 mmol⋅L^-1^ and above 6 mmol⋅L^-1^. These outcomes need to be analyzed in context with the conclusions of the observational and experimental studies in high-level athletes showing greater efficiency with the so-called polarized distribution (∼70–80% <2 mmol⋅L^-1^ and 10–20% >4 mmol⋅L^-1^) in running (Billat et al., 2001), rowing (Fiskerstrand and Seiler, 2004), cycling (Neal et al., 2013), and cross-country skiing (Tønnessen et al., 2014). However, relative to the polarized model, the polarized training zones in our study appeared to be moved upward: the fastest groups performed a lower proportion of SI (>4–6 mmol⋅L^-1^) training and higher MHI and EI proportions. These results agree with those of Arroyo-Toledo et al. (2013), who observed in young regional level swimmers a smaller performance improvement in 100 m following a traditional periodization (mean training volume of 23 km per week and an intensity distribution of 69% <2 mmol⋅L^-1^, 25% 2–4 mmol⋅L^-1^, and 6% >4 mmol⋅L^-1^) compared with a reverse periodization (weekly volume of 16 km; intensity distribution 49, 33, and 18%) (0.4 vs. 6.9%, respectively). In summary, 88% moderate-to-heavy intensity, 8% severe intensity and 4% extreme-intensity training for sprint swimmers, and 89%/7%/4% for middle-distance swimmers, with about the half performed below 2 mmol⋅L^-1^, reflects a good compromise in swimming between technical efficiency, sizeable physiological adaptations and management of training stress.

### Training Profiles – Olympic Quadrennials and Their Seasons

Olympic quadrennials and the seasons of the Olympic cycle were represented differently in the training profiles. These differences cannot easily be explained by the (scheduling of) competition dates as they varied considerably over the 20 years of the study. The variations more likely reflect the evolution in the training approaches of the coaches who trained the participants. Concerning the traditional methodological streams, a few authors have proposed a periodization model at the scale of a single Olympic quadrennial (Maglischo, 2003; Platonov, 2006; Lyakh et al., 2014). For instance, Platonov suggested that an annual periodization composed of two to three macrocycles would be appropriate for Olympic and World Championship seasons, and that a multi-cycle periodization (4–7 macrocycles per year) could be employed in intermediate seasons (e.g., continental or regional championships) (Platonov, 2006). Maglischo (2003) advocated an increase in volume and intensity during the years with Olympic Games and World Championships. However, the results of the present work do not agree with these assertions. For sprint swimmers, the most frequent and successful patterns in Olympic and World Championships seasons showed a medium total training load and progression from moderate-to-heavy to severe to extreme intensity training from the first to the second macrocycle. In addition, during pre- and post-Olympic seasons, we observed higher training volumes at moderate to heavy intensities [La]_b_ ≤ 4 mmol⋅L^-1^ in sprinters, and higher dryland workout volumes in middle-distance swimmers, suggesting these years of the Olympic quadrennial should be dedicated to developing less specific fitness.

The increased intensity during high-stakes competitive seasons (Olympic Games, World Championships) has been observed among world-class endurance athletes on a yearly scale (Tønnessen et al., 2014, 2015). As the major competitions of the season approach, the specificity and intensity of the training increases and non-specific training decreases. For the sprint swimmers in our analysis, it is worth noting that the Stable Flat macrocycle pattern group was mostly represented by the September 1996–September 2000 quadrennial, whereas the group corresponding to a pattern of Long macrocycles was mostly represented by the most recent quadrennials. We assume that the periodization methods presented over the last 15 years in methodological (Platonov, 2006) and sports science (Seiler and Kjerland, 2006) literature, as well as the hard-earned experiences of leading coaches communicated within the coaching community, have prompted changes in coaching practices.

### Training Profiles – Macrocycle Duration and Shape

A 25-weeks study period corresponds roughly to two typical macrocycles of 12–16 weeks that precede either the national selection or the major international (summer) championships. Over the study period, 47% of the swimmers achieved their best performance in the first macrocycle (typically leading to the national selection trials) and 53% in the second macrocycle (leading to major international competition). In the fastest sprint group, the second macrocycle lasted approximately 14–15 weeks. Training plans for some other endurance sports (Rønnestad et al., 2014; Tønnessen et al., 2014) have similar cycles or longer. In middle-distance track-and-field events (Tønnessen et al., 2014; Tønnessen et al., 2015), triathlon, and cycling (Rønnestad et al., 2014), the macrocycles typically last 12–14 weeks or more. Long cycles induce the cumulative effects in both physiological capabilities and physical/technical abilities resulting from long-term athletic training (Platonov, 2006; Lyakh et al., 2014; Issurin, 2016). There is evidence that the time course of physiological adaptations in training cycles lasts between 4 and 20 weeks (Holliday and Jeukendrup, 2012). Most of the metabolic, neuromuscular and cardiovascular adaptations to training that begin in the first 2–3 weeks culminate between 4 and 7 weeks and continue up to the 12th week or more. Consequently, a macrocycle of about 15 weeks seems effective to prepare swimmers for major competition.

The fastest sprint profile showed a waveform in the second macrocycle consisting of two progressive load peaks 10–11 and 4–6 weeks before competition. Training in the several weeks before a major competition clearly influences performance, with a positive impact of the general preparation mesocycle (i.e., weeks 9–11 prior to competition) (Hellard et al., 2017). In the fastest profile, these two peaks were separated by a load decrease. Conversely, the slowest training profile was characterized by a stable evolution with little variability. The observed wave periodization is similar to that described for models in the methodological literature (Maglischo, 2003; Platonov, 2006; Lyakh et al., 2014) and in the scientific and technical reports describing the training of Olympic and world-class sprint swimmers (Pyne and Touretski, 1993; Barnier, 2012). In these models, a phase devoted to developing strength and aerobic endurance is planned 8–11 weeks before the major competition. This phase is followed by the development of more specific qualities (maximal aerobic power, maximal anaerobic lactic power, and race pace intensity training), generally 4–6 weeks before the competitive period (Pyne and Touretski, 1993; Maglischo, 2003).

The studies on strength building (Kraemer and Ratamess, 2004) and the methodological recommendations on periodizing training loads (Issurin, 2016) argue that undulating periodization, alternating phases of volume, intensity and recovery provides an effective stimulus (Kraemer and Ratamess, 2004). As the body adapts rapidly to a specific type of physiological demand, volume and intensity changes are needed to ensure ongoing adaptation and progress (Maglischo, 2003; American College of Sports Medicine [ACSM], 2009).

### Training Profiles – Training Volumes, Variation in Total Training Load

The fastest profile among the sprint swimmers showed medium variations in training load, whereas the slowest one was characterized by low variability. The slowest profile in the middle distance swimmers showed high variability. Systematic (not excessive) variations in volume and intensity can yield greater long-term adaptations than training programs with a single and constant load (Issurin, 2016). Compared with the linear periodization method associated with long macrocycles (18–24 weeks or more), which are characterized by progressively increasing training loads followed by the stabilization that precedes the load decrease in competitive periods, the training of world-class endurance cyclists (Schumacher and Mueller, 2002), triathletes (Mujika, 2014) kayakers (Issurin, 2016), and swimmers (Maglischo, 2003; Barnier, 2012; Vergnoux, 2014) is characterized by shorter macrocycles made up of several 2–5 weeks mesocycles focused on the simultaneous development of two to three priority qualities, interspersed with short periods of reduced training (Maglischo, 2003). These periodization practices are close to the so-called multi-targeted block periodization method (Issurin, 2016), which consists of three types of block mesocycles: accumulation (development of endurance), transmutation (acquisition of specific technical motor skills) and realization (taper, specific preparation for competitive events), all of similar duration (2–4 weeks). The aim of this method is to develop energetic qualities and promote efficient transfer of general adaptations (strength and aerobic endurance) to specific adaptations (anaerobic endurance) with minimal risk of overtraining (García-Pallarés et al., 2010; Issurin, 2016). The effectiveness of this method was experimentally confirmed in world-class kayakers (García-Pallarés et al., 2010), well-trained cyclists (Rønnestad et al., 2014), as a 12-week block periodization yielded greater improvements in strength, maximal power output (MPO), power output at blood lactate concentrations of 2–4 mmol⋅L^-1^ and performance than traditional linear periodization. In summary, in macrocycles of about 15 weeks, swimmers, coaches and sports scientists should consider periodization involving two to three mesocycles, each lasting from 3 to 5 weeks interspersed with recovery periods (1–2 weeks).

### Training Profiles – Progressivity

Our results support the positive association between a progressive load increase from the first macrocycle to the second and sprint performance. The fastest profile showed progressive increases from one macrocycle to another and even within the last macrocycle. Conversely, in both sprint and middle-distance swimmers, the slowest profiles showed load decreases between the first and second macrocycles. The conceptual support for the principle of progressivity was inspired by the pioneering theory of Selye (1976) (the general adaptation syndrome), which described the adaptation to stress in three phases: (1) alarm, (2) adaptation/resistance, and (3) exhaustion. After an acute response in the first phase, adaptation (and performance) increases in the second phase but can stagnate in the third phase if the training stimulus remains constant. Training theorists have argued that the progressive nature of a training load facilitates a gradual increase in the training stimulus (Platonov, 2006; Dantas et al., 2010; Lyakh et al., 2014). Adaptive training processes are thus engaged because a stimulus of greater magnitude is induced (Kraemer and Ratamess, 2004; American College of Sports Medicine [ACSM], 2009). Progressive overloading should be introduced gradually into a program, and the swimmer should have sufficient time to adapt before coaches impose a new training load increment. The ACSM recommends that changes in total strength training volume (reps, sets, and load) be in increments of 2.5–5.0% per week to avoid overtraining. Although few studies have directly demonstrated the effectiveness of an increasing training load compared with a constant or decreasing load in endurance sports, Dantas et al. (2010) indicated that traditional models based on progressive increases in load and intensity were most effective. Moreover, experimental studies suggest that the greatest improvements in performance and physiological measures are associated with the progressive nature of the training load. For instance, cyclists who made the most progress (increased 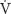
O_2_ max and power output at 2 mmol⋅L^-1^) after a block-type training program had more pronounced increases in volume and higher intensity than cyclists who followed a traditional training program (Rønnestad et al., 2014). A progressive increase in the training load over the season, either through an increase in TTL when it varies in a uniform way, or through the stabilization of TTL associated with a progressive increase in specificity and intensity, is a methodological principle most likely linked to the progression in performances. Clearly, swimming coaches should consider increasing the low- and high-intensity training loads from the first to the second half of the approximate 6-month (summer or winter) season leading to major national or international competitions.

### Take Home Message

For sprinters, coaches must consider the effectiveness of a progressive total training load from within and between each macrocycle until the beginning of the taper. Training load peaks should be located 7, 6, and 5 weeks before major competitions. For both sprint and middle-distance swimmers, it is advisable to avoid a large decrease in total training load between macrocycles and excessive training at swimming speeds corresponding to a blood lactate concentration between 4 and 6 mmol⋅L^-1^.

### Limitations

The end of the study period in 2012 is one of the limitations of this research. A recent review of the scientific and technical literature (Hawke, 2010; Barnier, 2012; Vergnoux, 2014; Vandenbogaerde et al., 2019) gives a more actual description of training in the last decade. In the period 2010–2018, the training of middle-distance swimmers (200–400 m) has been characterized by high volume, long continuous macrocycles, priority given to aerobic and threshold training, a short taper, and strong general physical preparation. The distribution of intensities has been characterized by a predominantly aerobic distribution, with 55–70% below [La]_b_ ≤ 2 mmol⋅L^-1^, and 30–40% between [La]_b_ ≤ 2 mmol⋅L^-1^ and [La]_b_ ≤ 4 mmol⋅L^-1^. In sprint swimming (50–100 m), the literature reveals two models in champion sprinters and Olympic medalists, with the first showing a high annual training volume (∼2,000–2,500 km) with a consistent proportion of aerobic and threshold training below [La]_b_ ≤ 4 mmol⋅L^-1^ (about 90% of the total training time), and the second showing a lower volume (∼1,000–1,500 km) composed of a high intensity training (volume below [La]_b_ = 2 mmol⋅L^-1^ higher than 70% and volume [La]_b_ = 4 mmol⋅L^-1^ tending toward 15%). This swimming training is associated with daily dryland training focused on increasing maximum strength, power and training capabilities.

Secondly, the training load quantification was based exclusively on lactate measurements, which provides only a partial view and is subject to measurement error. Thirdly, several covariates besides the training load may impact performance (Bourdon et al., 2017). A swimmer’s basal fitness, current life constraints, nutrition, recovery measures, psychological responses and technical quality during training are some of the potential unmeasured confounding factors. Also, as our analyses were based on the marginal associations between the different profiles in the load changes and various training characteristics (distribution, progressivity, and variability), the association between the profile characteristics and performance efficiency cannot be inferred to be causal. A future study should statistically assess the links between these training parameters and performance improvement.

## Conclusion

We identified training profiles from a 20-year cohort of elite French swimmers and characterized them in terms of relative performance, season within the Olympic quadrennial, Olympic quadrennial and training contents. Training practices have clearly evolved over the years, and advances in scientific and empirical knowledge may have contributed to changes in practices. We note that pre- and post-Olympic swimming seasons seem to be dedicated to developing less sport-specific fitness, while (particularly for sprinters) the Olympic Games and World Championship seasons show a progression from low- to high-intensity training from the first to the second macrocycle. In sprinters, the fastest group showed a progressive load increase from the first to the second half of a 6-month period leading to major competitions. Conversely, in both sprint and middle-distance swimmers, the slowest groups showed load decreases between the two macrocycles. A traditional macrocycle of about 15 weeks, comprising two to three mesocycles, each lasting about 4–6 weeks interspersed with recovery periods, seems to be a widely established practice for preparing swimmers for major competitions. Finally, swimming training distributions were 88–89% at ≤4 mmol⋅L^-1^ (with half <2 mmol⋅L^-1^) and 4% at >6 mmol⋅L^-1^, which differs from the more polarized distributions reported for other endurance sports. A possible explanation for this difference could be the higher technical swimming efficiency in the 2 to ≤4 mmol⋅L^-1^ training intensity zone.

## Author Contributions

PH provided the funding, collected data, and he has written the manuscript. MA-F developed statistical methods and has co written the manuscript. GL developed and applied the statistical methods. RP performed the figures and collected data. J-FT provided the funding. DP served as director of the research. IM reread the manuscript.

## Conflict of Interest Statement

The authors declare that the research was conducted in the absence of any commercial or financial relationships that could be construed as a potential conflict of interest.
